# Hyperreflective Membrane at the Vitreoretinal Interface in Diabetic Macular Edema: A Finding in Ultra-High-Resolution Optical Coherence Tomography

**DOI:** 10.1167/tvst.11.9.21

**Published:** 2022-09-23

**Authors:** Iori Wada, Shintaro Nakao, Mitsuru Arima, Keijiro Ishikawa, Muneo Yamaguchi, Yoshihiro Kaizu, Haruka Sekiryu, Kenichiro Mori, Kohei Kiyohara, Atsunobu Takeda, Tatsuro Ishibashi, SriniVas R. Sadda, Koh-Hei Sonoda

**Affiliations:** 1 Department of Ophthalmology, Graduate School of Medical Sciences, Kyushu University, Fukuoka, Japan; 2 Department of Ophthalmology, National Hospital Organization, Kyushu Medical Center, Fukuoka, Japan; 3 Clinical Research Institute, National Hospital Organization, Kyushu Medical Center, Fukuoka, Japan; 4 Doheny Image Reading Research Lab, Doheny Eye Institute, Los Angeles, California, USA

**Keywords:** posterior hyaloid membrane, VEGF, diabetic retinopathy, inner limiting membrane, vitreous

## Abstract

**Purpose:**

Detecting subtle vitreoretinal interface (VRI) findings, such as a posterior hyaloid membrane, is difficult with conventional retinal imaging. We compared ultra-high-resolution spectral domain optical coherence tomography (UHR-SD-OCT) with standard-resolution OCT (SD-OCT) for the imaging of VRI abnormalities in diabetic retinopathy (DR).

**Methods:**

This prospective cross-sectional study included 113 consecutive patients (91 patients with diabetes and 22 healthy controls). The VRI was evaluated, and the results were compared between the conventional SD-OCT and UHR-SD-OCT images. VRI findings were also investigated before and after internal limiting membrane peeling during vitrectomy for proliferative DR.

**Results:**

A total of 159 eyes (87.4%) of 91 patients with diabetes were analyzed. UHR-SD-OCT could detect a hyperreflective layer at the VRI, in which en face OCT showed a membrane-like structure, termed the hyperreflective membrane (HRMe). The preoperative HRMe could not be detected in all patients with proliferative DR who underwent internal limiting membrane peeling during vitrectomy. Although the HRMe did not correlate with the DR stage, eyes with diabetic macular edema (DME) (64.5%) showed a significant HRMe with UHR-SD-OCT more frequently than those without DME (35.8%) (*P* = 0.005).

**Conclusions:**

UHR-SD-OCT can detect the HRMe at the VRI in DR eyes, particularly in eyes with DME. The HRMe may present a thickened posterior hyaloid membrane that contributes to DME development.

**Translational Relevance:**

UHR-SD-OCT detects slight changes in the VRI in DR eyes. In the future, it may help to elucidate the mechanism of DME formation.

## Introduction

With aging, the vitreous normally detaches from the retina.^1^^,^^2^ However, incomplete or abnormal posterior vitreous detachment (PVD) occurs in some eyes, resulting in an abnormal vitreoretinal interface (VRI).^3^ VRI abnormalities play important roles in the pathogenesis of diabetic retinopathy (DR).^4^ Histological studies have shown posterior hyaloid membrane thickening (also called thickened vitreous cortex) in diabetic macular edema (DME).^5^^,^^6^ A large number of cellular elements have been observed on the vitreous side of the internal limiting membrane (ILM) in patients with DME.^7^^,^^8^ These observations suggest that VRI abnormalities, including the epiretinal membrane (ERM) and posterior membrane thickening, contribute to DME pathogenesis.^3^^,^^9^ However, current imaging modalities are unable to estimate posterior membrane thickening in DME owing to insufficient resolution.

DME is a major cause of vision loss in combination with DR, whereby breakdown of the blood–retinal barrier occurs with concomitant leakage of plasma and lipids in the macula.^10^^,^^11^ Both chemical and mechanical factors are involved in DME formation.^12^ Chemical factors, such as vascular endothelial growth factor, are upregulated by ischemia, oxidative stress, or inflammation, which increases vascular permeability and causes DME.^12^ Mechanical factors have also been proposed to play important roles in the development and progression of DME, such as VRI alterations with vitreomacular traction, ERM formation, and a thickened posterior hyaloid membrane. Various studies have reported the effectiveness of PVD and peeling of the ERM with or without ILM in vitrectomy for DME.^5^^,^^13^^–^^17^

Optical coherence tomography (OCT), a fast convenient diagnostic tool for the quantitative measurement and mapping of macular thickness, is the gold standard for DME diagnosis.^18^ The resolution of conventional OCT is 5 to 8 µm, which is sufficient for observing the ERM but not for more subtle VRI alterations, such as mild posterior hyaloid membrane thickening.

Recently, several groups have developed ultra-high-resolution OCT with axial image resolutions of approximately 3 µm.^19^^–^^21^ Previous studies have compared ultra-high-resolution spectral domain OCT (UHR-SD-OCT) with standard-resolution SD-OCT for imaging macular diseases.^22^^,^^23^ However, the VRI of DR and DME eyes using UHR-SD-OCT has not been examined. We also recently developed a new clinical UHR-SD-OCT (Bi-µ, Kowa, Tokyo, Japan) system using an original average technique called the “A-scan matching algorithm” with a resolution of 2 µm.^24^ In this study, we compared UHR-SD-OCT with SD-OCT for VRI imaging.

## Methods

### Ethics Statement

This study was approved by the Institutional Ethics Committee of Kyushu University Hospital (Protocol No. 26012, UMIN000017473) and was performed in accordance with the tenets of the Declaration of Helsinki. Written informed consent was obtained from all the patients after providing a detailed explanation of the study.

### Patient Population

This prospective study included 91 consecutive patients with diabetes mellitus who visited the Kyushu University Hospital between November 2016 and December 2019. We excluded eyes with any other ocular disease that may lead to VRI alterations (e.g., retinal vascular occlusion, age-related macular degeneration, and glaucoma). Patients who had previously undergone vitrectomy were also excluded. Twenty-two healthy individuals were included in the study as controls.

### Ophthalmic Examination

At each follow-up visit, patients underwent a complete ophthalmic examination, which included slit-lamp biomicroscopy, dilated fundoscopy, fundus photography, SD-OCT (Cirrus HD-OCT model 5000; Carl Zeiss Meditec, Inc., Dublin, Ireland), and UHR-SD-OCT (Bi-µ, Kowa). Another SD-OCT device (Heidelberg retina angiograph-OCT; Heidelberg Engineering, Heidelberg, Germany) was used only in cases where fluorescein angiography imaging was required. The central macular thickness (CMT) was measured using OCT as the average thickness of the central 1-mm thickness map measurement area.

### UHR-SD-OCT

UHR-SD-OCT was used to obtain cross-sectional images (B-scans) of the retinal structures horizontally with an axial resolution of 2.0 µm and image acquisition speed of 37,000 single axial scans (A-scans)/s. Each A-scan had a depth of 2.6 mm comprising 2000 pixels that allowed a digital depth sampling of 1.3 µm/pixel. Each B-scan spanned 30° or approximately 9 mm and consisted of 2000 A-scans.^24^ In healthy individuals, the right eye was imaged using UHR-SD-OCT. Supplemental Table 1 shows the comparison between UHR-SD-OCT and conventional SD-OCT. The most remarkable difference was observed in the optical resolution (UHR-SD-OCT vs. conventional SD-OCT: 2 µm vs. 5–7 µm).

### Evaluation of Hyperreflective Membrane (HRMe) Presence Using OCT Images

Two independent retina specialists (YK, HS) evaluated the presence of the HRMe at the VRI in two horizontal OCT images (conventional SD-OCT [Cirrus HD-OCT model 5000, Carl Zeiss Meditec, Inc.] and UHR-SD-OCT [Bi-µ, Kowa]). A third observer (IW) adjudicated on cases where the evaluations of the two observers were different. The kappa coefficient was 0.86 (95% confidence interval, 0.75–0.98; *P* < 0.0001) for the evaluation of the HRMe.

### Vitrectomy

Four patients with proliferative DR (PDR) with vitreous hemorrhage (two cases) or tractional RD (two cases) underwent surgery with pars plana vitrectomy using a 25G system and posterior hyaloid dissection, ERM excision when present, and ILM peeling (diameter ≥3000 µm), assisted in all cases with brilliant blue G staining (Coomassie BBG 250; Sigma-Aldrich Co., St Louis, MO). Cataract extraction with intraocular lens implantation was performed in all cataractous cases.

### Statistical Analyses

All statistical analyses were performed using a commercial software package (JMP Pro software version 12.0; SAS, Inc., Cary, NC). Descriptive statistics included mean, standard deviation, median, range, and percentages, where appropriate. Correlations between two variables were analyzed using analysis of variance, the χ^2^ test, multiple logistic regression analysis, and the Wilcoxon rank-sum test: sex, age, CMT, and changes in OCT parameters. All associations were considered statistically significant at a *P* value of less than 0.05.

## Results

Participants’ characteristics are presented in Table. Ninety-one patients with diabetes mellitus were recruited in this study and 159 eyes (87.4%) were analyzed (mean age, 60.7 ± 13.2 years; 49 male and 42 female; 9 patients with non-DR, 42 with mild or moderate nonproliferative DR [NPDR], 11 with severe NPDR, and 29 with PDR). Of the 159 eyes, 128 had no DME and 31 had DME. Of the 25 patients with treatment-naïve DME, 15 were male and 10 were female; 10 eyes had mild or moderate NPDR, 5 had severe NPDR, and 10 had PDR (Supplemental Table 2). The average age of the patients was 57.9 ± 12.1 years (range, 34–73 years) and the average CMT was 474.7 ± 128.9 µm. Twenty-two eyes of 22 healthy individuals (mean age, 62.7 ± 15.9 years; range, 43–87 years) were also analyzed (Table).

**Table.** Demographic Characteristics of the Study Participants

Characteristics	Healthy	NDR	Mild to Moderate NPDR	Severe NPDR	PDR	*P* Value
No. of patients (%)	22	9 (9.5)	42 (45.9)	11 (12.2)	29 (32.4)	–
No. of analyzable eyes	22	15	64	22	58	–
Age (years)	62.7 ± 15.9	50.7 ± 17.1	60.1 ± 13.3	56.0 ± 13.0	61.4 ± 12.0	0.32
Duration of diabetes mellitus (years)	–	7.6 ± 1.8	8.2 ± 1.5	11.8 ± 1.5	7.4 ± 1.5	0.048
HbA1c (%)	–	3.5 ± 2.2	18.4 ± 10.3	12.0 ± 10.5	12.2 ± 12.4	0.23
Incidence of treatment (0)	0 (0)	0 (0)	19 (45.2)	11 (100)	27 (93.1)	< 0.001
HRMe (%)	6 (27.2)	3 (33.3)	17 (40.5)	4 (36.4)	11 (37.9)	0.98


HbA1c, glycated hemoglobin, percent of total hemoglobin.

Wilcoxon rank sum test or χ^2^ test.

**Figure 1.**
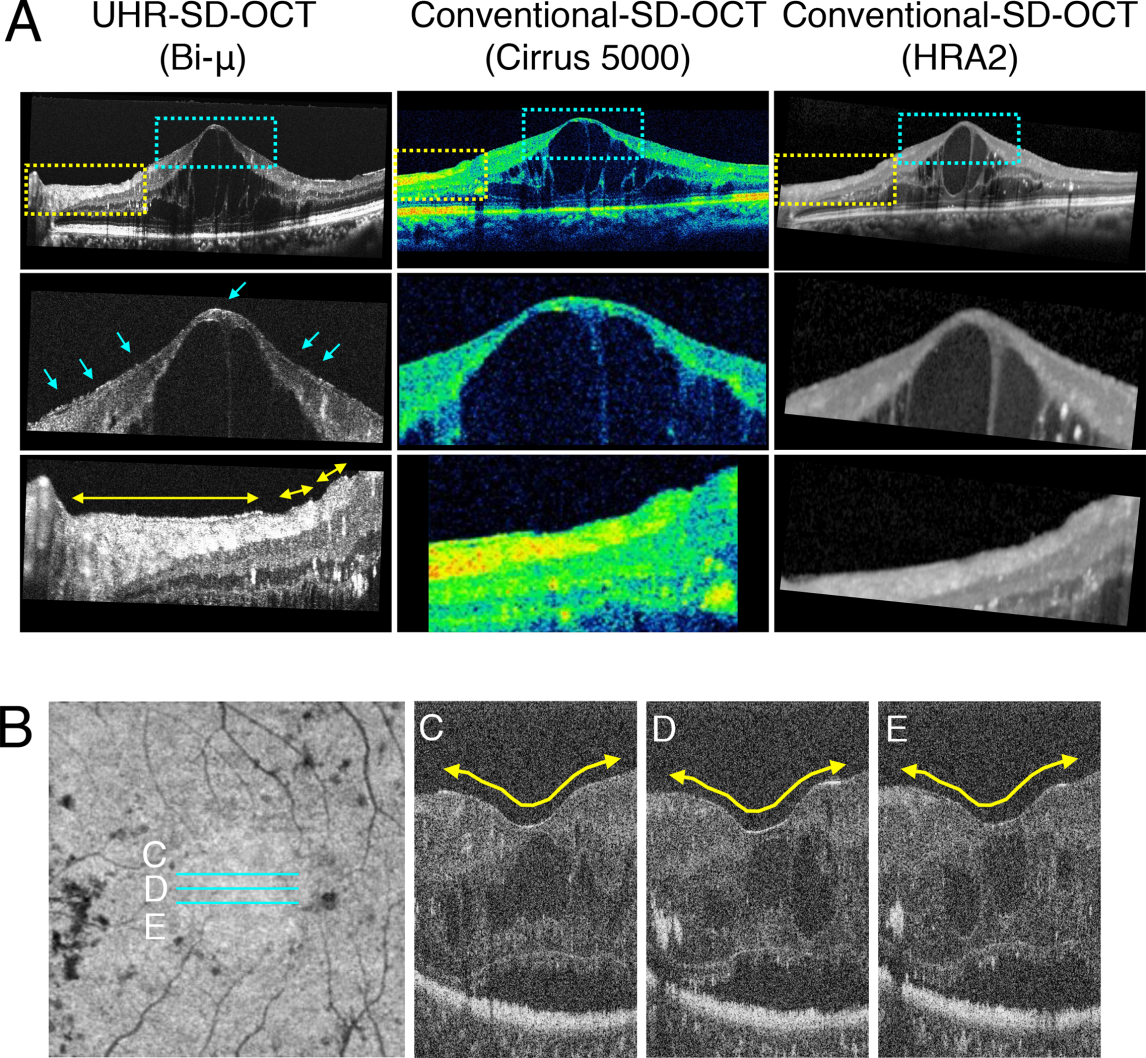
Comparison between UHR-SD-OCT and conventional spectral domain OCT (SD-OCT) for VRI imaging. (A) Representative images of the HRMe on VRI of Bi-µ (UHR-SD-OCT), Cirrus 5000 (Conventional-SD-OCT), and HRA2 (Conventional-SD-OCT) in a 59-year-old woman with DME. The *blue* and *yellow* dotted squares indicate magnified OCT images in *middle* and *lower images*, respectively. *Blue arrows (middle left image)* and the *yellow arrow (lower left image)* show the HRMe. (B) *Yellow arrows* show the HRMe at three different consecutive sections (*blue lines*; C–E) by en face UHR-SD-OCT image (in a 49-year-old man with DME).

**Figure 2.**
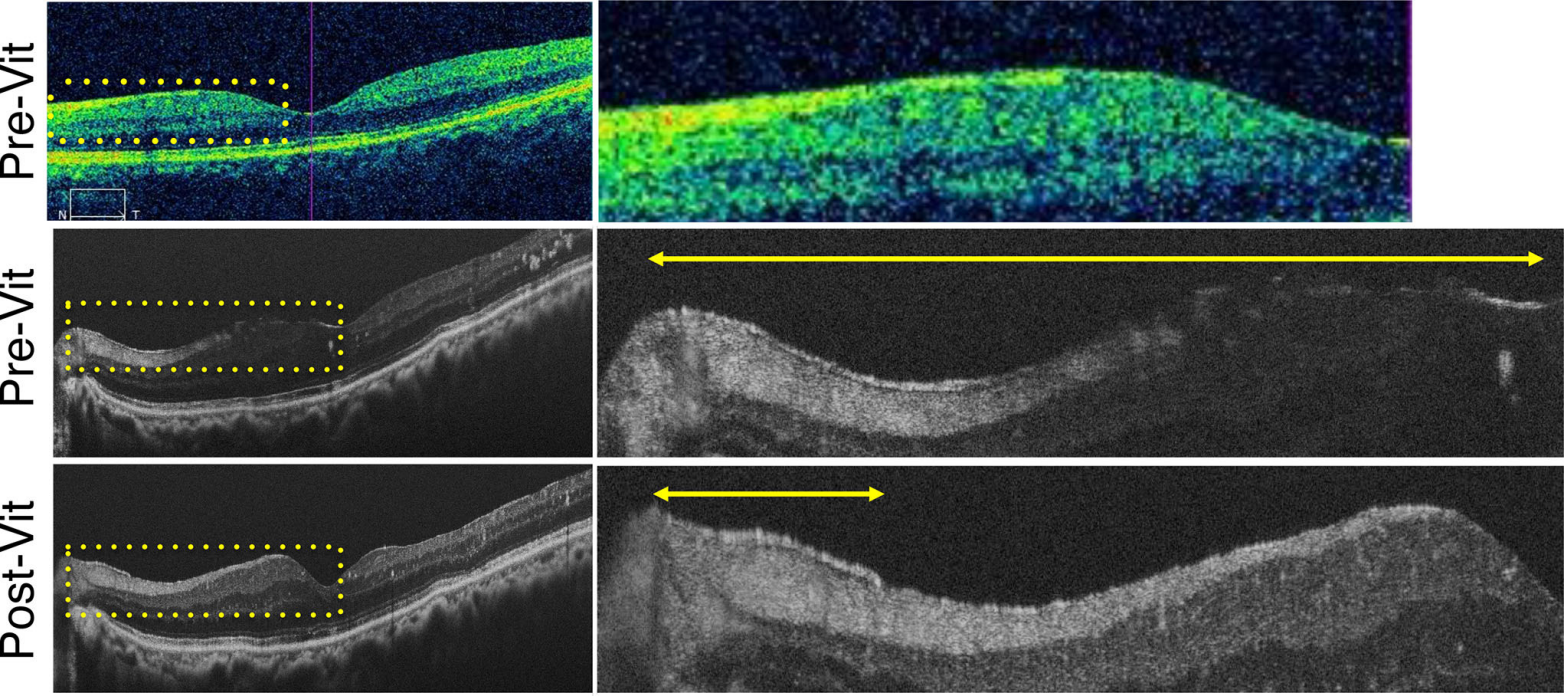
Representative images of the HRMe before and after vitrectomy with ILM peeling in a 66-year-old man with PDR (left eye). (*Top*) Conventional spectral domain OCT (SD-OCT; Cirrus 5000) shows no apparent ERM before vitrectomy. (*Middle*) UHR-SD-OCT shows the HRMe in the area between the optic disc and fovea before vitrectomy. (*Bottom*) UHR-SD-OCT shows disappearance of the HRMe in the same area after vitrectomy. *Yellow dotted squares* indicate magnified OCT images in the right images. *Yellow arrows* show the HRMe.

First, VRI findings between UHR-SD-OCT (Bi-µ) and conventional SD-OCT (Cirrus HD-OCT model 5000) were compared in all subjects. A hyperreflective layer was observed on UHR-SD-OCT that could not be detected by conventional SD-OCT in some DR cases (Fig. 1A). Next, the en face image of UHR-SD-OCT was used to investigate whether the hyperreflective layer was a “membrane”-like structure at the VRI (Fig. 1B). En face UHR-SD-OCT confirmed the membrane-like structure at the VRI, henceforth termed the HRMe. Moreover, to determine whether the HRMe was an artifact, we compared UHR-SD-OCT images before and after vitrectomy in four patients with PDR. The HRMe disappeared after vitrectomy with ILM peeling (Fig. 2), suggesting that it was a subtle VRI abnormality and possibly a mild thickening of the posterior hyaloid membrane.

**Figure 3.**
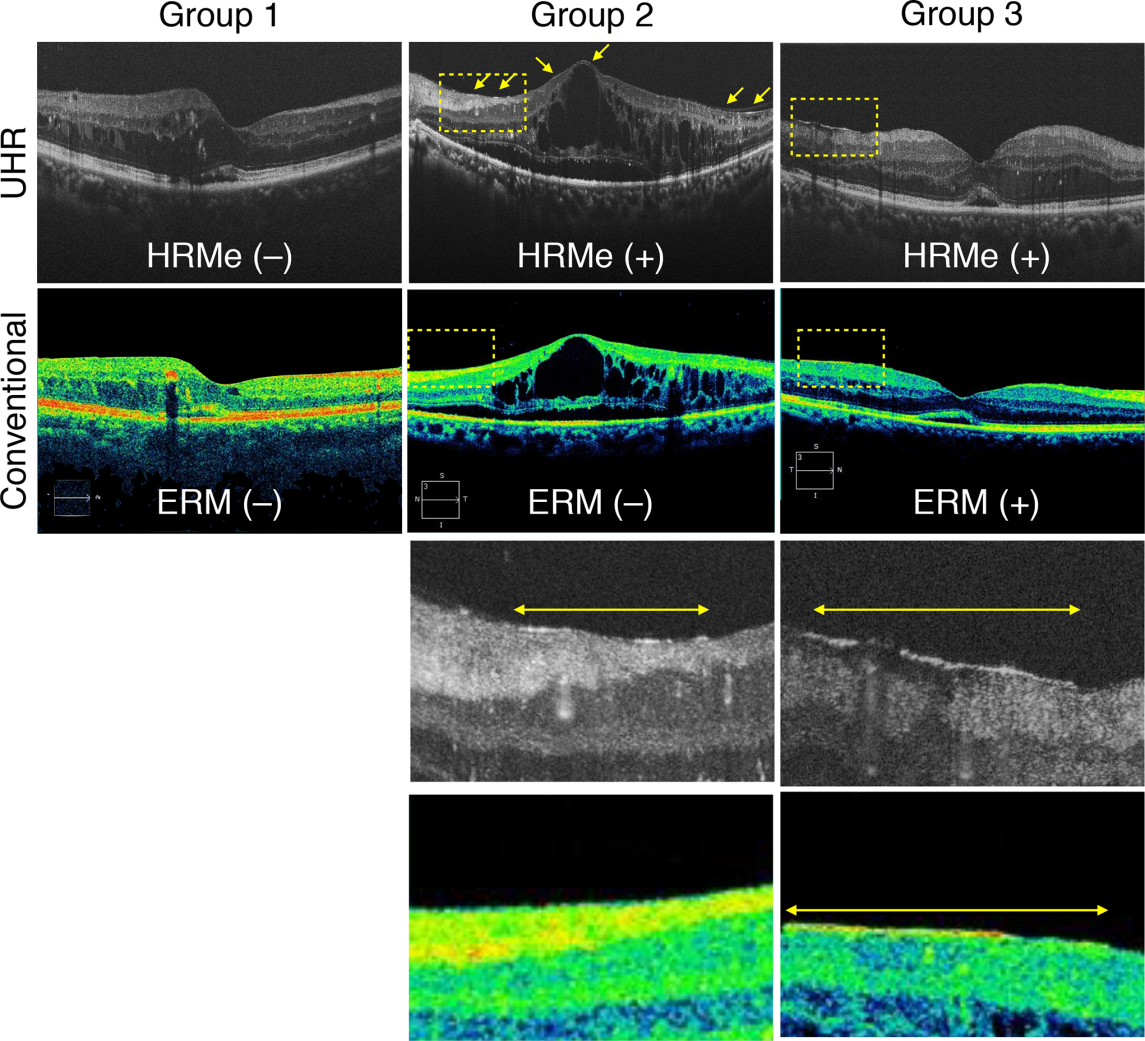
The classification of VRI in UHR-SD-OCT and conventional spectral domain OCT (SD-OCT). Group 1: A representative image of a 63-year-old woman with DME. The HRMe (−) in UHR-SD-OCT. ERM (−) in SD-OCT. Group 2: A representative image of a 46-year-old man with DME. The HRMe (+) in UHR-SD-OCT. ERM (−) in SD-OCT. Group 3: A representative image of 71-year-old woman with DME. The HRMe (+) in UHR-SD-OCT. ERM (+) in SD-OCT. *Yellow dotted squares* indicate magnified OCT images in the lower images. *Yellow arrows* and *lines* show the HRMe.

Membranes at the VRI were classified into the following three groups: group 1, no membrane structure could be detected using either UHR-SD-OCT or conventional SD-OCT; group 2, the HRMe was detected by UHR-SD-OCT without apparent ERM and was not detected by conventional SD-OCT; and group 3, the membrane was detectable using UHR-SD-OCT and conventional SD-OCT (Fig. 3).

Next, 35.8% of eyes without DME showed the HRMe, whereas 64.5% of eyes with DME showed the HRMe, indicating a higher HRMe detection rate in eyes with DME (*P* = 0.005). However, the presence of an HRMe was not correlated with age, sex, diabetes duration, or DR stage.

Moreover, the correlation between the HRMe and macular thickness in DME was investigated. CMT was significantly thicker in patients with DME in whom membrane was detected than in those without membrane (527.7 ± 128.4 µm vs. 409.7 ± 125.3 µm, *P* = 0.03). In contrast, for healthy eyes, an HRMe was observed in six eyes (27.3%), and the CMT was similar between those with an HRMe (257.6 ± 14.6 µm) and without an HRMe (254.4 ± 18.3 µm).

## Discussion

This prospective study compared UHR-SD-OCT with standard-resolution SD-OCT for VRI imaging in patients with DR and DME. We noticed a hyperreflective finding, the HRMe, that was detectable by UHR-SD-OCT, but not by conventional SD-OCT. Although the HRMe was also observed in some healthy subjects (27.2%), this finding was more frequent in patients with DME. In DME cases with an HRMe, the CMT was significantly greater than that in those without an HRMe. A subclinical abnormal VRI, which is not recognized as an ERM on conventional OCT, might contribute to DME pathogenesis.

Several groups have developed UHR-SD-OCT and evaluated its advantages. Ko et al.^22^^,^^23^ compared UHR-SD-OCT with standard-resolution SD-OCT for imaging various macular diseases, including macular holes, macular edema, and age-related macular degeneration. These previous studies reported that UHR-SD-OCT enhances the visualization of intraretinal architectural morphology relative to standard-resolution OCT (SD-OCT).^22^^,^^23^ Witkin et al.^25^ examined the VRI in 19 eyes of 18 patients with lamellar holes using UHR-SD-OCT and reported that, in the majority of eyes, the ERM was visualized on UHR-SD-OCT. However, there have been no reports examining the VRI in patients with DR using UHR-SD-OCT. Our study showed that most eyes with DME (64.5%) demonstrated an HRMe on UHR-SD-OCT, but no evidence of a similar finding or ERM was observed on conventional SD-OCT. Although the thickness of the ERM has been reported to be 10 to 20 µm,^26^ a pathology study has shown that the posterior vitreous membrane thickness is less than 2 µm.^27^ The resolution of our UHR-SD-OCT was 2 µm, whereas that of the conventional SD-OCT used in this study was 5 to 8 µm. Conventional SD-OCT can detect ERM; however, it is presumed that the detection of a very subtle ERM or the posterior vitreous membrane would be difficult. The ERM can be observed with conventional OCT or fundus examination, but posterior vitreous membrane thickening that can be visualized with triamcinolone during surgery cannot be identified with fundus examination or OCT.^28^^,^^29^ Therefore, an HRMe that can be imaged only with UHR-SD-OCT might represent thickening of the posterior vitreous membrane or a very thin ERM. However, sequential observation is necessary to determine whether an HRMe is a precursor of ERM. Furthermore, it is known that both the posterior vitreous membrane and ERM are primarily comprised of collagen, although the constituent cells may differ.^30^^,^^31^ Therefore, it may be difficult to distinguish between an HRMe and ERM on UHR-OCT, except in terms of thickness.

This study showed that the baseline CMT in patients with DME with an HRMe was significantly thicker than that in those without an HRMe. This result might support the widely accepted concept that both chemical and mechanical factors are relevant to the development of DME.^32^ Some previous studies have shown many cells including vascular, neural cells, and myofibroblasts in the posterior hyaloid in patients with DR.^30^ Myofibroblasts positive for α-smooth muscle actin may induce cell contraction, resulting in gel contraction.^31^^,^^33^ Furthermore, these cells at the VRI can secrete vascular permeability cytokines, including vascular endothelial growth factor.^33^ However, we cannot exclude the possibility that VRI changes, such as an HRMe, are not a causal factor of DME, but rather an accompanying sequela.

In the present study, an HRMe was also observed in some healthy eyes. Our retrospective observation showed that the HRMe in healthy eyes was significantly shorter than in diabetic eyes (1.12 ± 1.07 mm vs. 2.25 ± 1.52 mm; *P* < 0.05). It is reported that posterior vitreous membrane thickening can be observed during vitrectomy in cases such as retinal detachment without diabetes.^29^ The average age of the healthy participants in this study was 62.7 years. Therefore, PVD may have occurred in many cases.^1^^,^^2^^,^^34^

This study had several limitations. First, the sample size was small. Second, some DR cases (12.6%) could not be analyzed because of insufficient image quality. This lack of quality may be due to poor fixation associated with poor visual acuity or vitreous hemorrhage, which may be associated with an abnormal VRI. Third, we included patients with previous treatment for DR (e.g., anti-vascular endothelial growth factor therapy or panretinal photocoagulation, despite no recent treatment). These therapies may also affect VRI status. Further studies are needed to investigate whether these treatments can affect the VRI findings evident on UHR-SD-OCT. Fourth, this study did not consider PVD. We cannot rule out the possibility that PVD may have influenced our results.

## Supplementary Material

Supplement 1
