# Supplementary material for: Hyperreflective Membrane at the Vitreoretinal Interface in Diabetic Macular Edema: A Finding in Ultra-High-Resolution Optical Coherence Tomography
Source: Transl Vis Sci Technol. 2022 Sep 23;11(9):21. doi: 10.1167/tvst.11.9.21 (PMC9520517; doi:10.1167/tvst.11.9.21)
Supplement: Supplement 1 [file tvst-11-9-21_s001.pdf]

Item		Bi-μ	HRA2	Cirrus 5000
Theory		SD-OCT	SD-OCT	SD-OCT
Light source		Broadband SLD	SLD	SLD
Center wavelength		860 nm	870 nm	840 nm
Optical axis (in tissue)	Optical resolution	2.0 μm	7.0 μm	5.0 μm
	Digital resolution	1.3 μm (2048 pixels)	3.9 μm (496 pixels)	1.9 μm (1024 pixels)
Scan range	horizontal	Max. 12 mm	55° / 16.5 mm (Wide module)	36 x 30°
	depth	2.6 mm	1.9 mm	2.0 mm
A-scan speed		80,000 / s	85,000 / s	27,000-68,000 / s

Supplemental Table 1 Comparison between UHR-SD-OCT and two conventional SD-OCT

Characteristics	Mild or moderate NPDR	Severe NPDR	PDR	*P*
Number of patients (%)	10 (40)	5 (20)	10 (40)	-
Number of analyzable eyes	13	7	11	-
Age (years)	59.8 ± 8.8	65.0 ± 8.6	51.0 ± 13.7	0.064
Duration of DM (years)	10.5 ± 8.1	16.3 ± 6.5	11.7 ± 5.5	0.36
HbA1c (%)	8.1 ± 1.3	6.7 ± 0.7	8.0 ± 1.3	0.044
CMT (μ m)	472.8 ± 123.0	448.3 ± 158.2	493.6 ± 110.6	0.54

(*Wilcoxon rank sum test*)

Supplemental Table 2 Patients with naïve DME
